# METTL16 Promotes Lipid Metabolic Reprogramming and Colorectal Cancer Progression

**DOI:** 10.7150/ijbs.105391

**Published:** 2025-07-24

**Authors:** Jie Li, Qian Luo, Minjie Lu, Chen Lu, Caihong Xu, Jie Ding, Tian Zhan, Jing Zhu, Mengsen Qian, Shuhui Lin, Lisha Chang, Juan Li, Keming Wang

**Affiliations:** 1 Department of Oncology, The Second Affiliated Hospital of Nanjing Medical University, Nanjing, Jiangsu, China.; 2 Department of Oncology, Taicang TCM Hospital Affiliated to Nanjing University of Chinese Medicine, Suzhou, Jiangsu, China.; 3 Department of Cardiothoracic Surgery, Zhongda Hospital, School of Medicine, Southeast University, Nanjing, Jiangsu, China.; 4 Department of Obstetrics and Gynecology, Nanjing Tongren Hospital, School of Medicine, Southeast University, Nanjing, Jiangsu, China.; 5 Department of General Surgery, Second Affiliated Hospital, Nanjing Medical University, Nanjing, Jiangsu, China.

**Keywords:** CRC, m6A, M16, metabolism

## Abstract

**Background:** Lipid reprogramming represents a pivotal stage in tumor progression. N6-methyladenosine (m6A), the most prevalent RNA modification in eukaryotic cells, plays a significant role in colorectal cancer (CRC) development, though its specific involvement in lipid reprogramming remains unclear.

**Methods:** Bioinformatics analysis of The Cancer Genome Atlas (TCGA) and Gene Expression Omnibus (GEO) databases revealed differential expression of METTL16 (M16), which was further validated through qRT-PCR and Western blotting in CRC tissues and cell lines. The impact of M16 on CRC proliferation, metastasis, invasion, and lipid reprogramming was evaluated using both *in vivo* and *in vitro* approaches. Regulatory mechanisms underlying M16's role in CRC progression were explored using immunofluorescence (IF) staining, RNA immunoprecipitation (RIP), MERIP assay, RNA pull-down assay, total m6A measurement, RNA stability assay, protein stability analysis, and luciferase reporter assays.

**Results:** Analysis results demonstrated a significant upregulation of the m6A methyltransferase METTL16 in CRC, closely associated with poor prognosis and abnormal lipid droplet accumulation. Functional assays revealed that M16 overexpression markedly promotes CRC cell proliferation, migration, and invasion both *in vitro* and *in vivo*, primarily by enhancing lipid reprogramming. Mechanistically, M16 induces m6A modification of TM7SF2 mRNA, stabilizing it via an IGF2BP1- and IGF2BP2-dependent pathway, thereby upregulating TM7SF2 expression and driving lipid reprogramming in CRC.

**Conclusion:** In conclusion, these findings highlight the critical role of the M16/m6A/TM7SF2 axis in lipid metabolic reprogramming in CRC, offering potential therapeutic targets for its treatment.

## Introduction

CRC is one of the most common malignant tumors of the digestive tract. It is estimated that annually there are approximately 1,926,000 new cases and 903,000 deaths worldwide 1. The subtle onset of CRC symptoms often results in late-stage diagnoses, complicating treatment and leading to suboptimal outcomes 2. Consequently, the identification of novel biomarkers and effective therapeutic targets for early detection and treatment is essential.

Energy metabolic reprogramming, a hallmark of cancer progression, is frequently dysregulated in tumors 3. Increasing evidence suggests that tumor progression is often accompanied by disruptions in lipid metabolism, including alterations in lipid uptake, synthesis, and hydrolysis 4. Lipid metabolic reprogramming influences membrane biosynthesis, energy storage, and the regulation of CRC cell homeostasis, thereby enhancing cellular proliferation, migration, and invasion 5,6. Additionally, lipids contribute to signal transduction by generating second messengers, such as those activating oncogenic pathways like RAS and RHO, or by secreting lipids into the tumor microenvironment to inhibit immune cell antitumor responses 7,8. These observations emphasize the critical role of lipid metabolism in CRC progression.

The most widespread internal RNA alteration in eukaryotic cells is m6A, playing a pivotal role in various RNA regulatory processes in tumor cells 9-11. The m6A modification is catalyzed by RNA methyltransferases (METTL3/14/16, etc.) that target RNA and m6A motifs, and this process can be reversed by RNA demethylases (ALKBH5, FTO). RNA-binding proteins (IGF2BP1/2/3, YTHDC1, YTHDF1/2/3, etc.) identify and bind to m6A motifs, thereby influencing RNA splicing, translocation, stability, and protein translation 12-14. Increasing evidence highlights the involvement of m6A modifications in various physiological and pathological processes, particularly in tumorigenesis, development, metastasis, treatment resistance, and recurrence 15,16. However, while methyltransferases such as METTL3 and METTL14 have been extensively studied, the role of METTL16 (M16) in CRC, particularly in lipid metabolism, remains underexplored.

This study demonstrates that M16 overexpression promotes lipid metabolic reprogramming and CRC progression. Mechanistically, M16-mediated m6A modifications enhance the stability of TM7SF2 mRNA *via* IGF2BP1- and IGF2BP2-dependent pathways, thereby facilitating lipid metabolic reprogramming and promoting CRC progression. These findings position M16 as a potential novel biomarker and therapeutic target for CRC.

## Materials and Methods

### CRC tissue specimens and clinical data

CRC samples and neighboring non-tumor tissues were collected from Nanjing Medical University's Second Affiliated Hospital. Detailed clinical data for the participants are provided in Supplementary Files. All participants provided informed consent prior to inclusion in the study, which was approved by the Ethics Committee of the Second Affiliated Hospital of Nanjing Medical University.

### Cell lines and transfection

The normal intestinal epithelial cell line FHC and CRC cell lines (SW480, HCT116, LOVO, SW620, DLD1) were sourced from the American Type Culture Collection. All cell lines were cultured in RPMI-1640 (Gibco, USA) or DMEM (Gibco, USA) supplemented with 10% fetal bovine serum (FBS) (CLARK, USA).

GenePharma (Shanghai, China) engineered and packaged shRNAs targeting M16, IGF2BP1, IGF2BP2, IGF2BP3, and TM7SF2, as well as overexpression plasmids, into lentiviral vectors for transfection. To establish stably transfected cell lines, CRC cells were transfected with shRNAs or plasmids using Lipofectamine 2000 (Invitrogen, USA), following the manufacturer's instructions. The shRNA sequences are listed in Supplementary Table S1.

### RNA Extraction and Quantitative Real-Time PCR (qRT-PCR)

Total RNA was extracted from CRC cells or tissues using TRIzol reagent (Invitrogen, USA), followed by phase separation with isopropanol and chloroform. RNA was reverse-transcribed into cDNA using the Evo M-MLV Reverse Transcription Kit (AG, China). To quantify mRNA expression levels, qRT-PCR was used using SYBR Green Pro Taq HS Premix (AG, China), with GAPDH as an internal reference. Supplementary Table S2 has a full description of primer sequences.

### Western blotting

Western blotting was performed as previously described 17. Antibodies used in this study included anti-M16 (ab252420, Abcam), anti-IGF2BP1 (22803-1-AP, Proteintech), anti-IGF2BP2 (11601-1-AP, Proteintech), anti-IGF2BP3 (14642-1-AP, Proteintech), anti-YTHDF1 (17479-1-AP, Proteintech), anti-YTHDF2 (24744-1-AP, Proteintech), anti-YTHDF3 (25537-1-AP, Proteintech), anti-YTHDC1 (14392-1-AP, Proteintech), and anti-TM7SF2 (12033-1-AP, Proteintech). Beta-actin (66009-1-lg, Proteintech) was used as the loading control.

### Immunohistochemistry (IHC) staining

Immunohistochemical (IHC) staining was performed as previously described 17. Antibodies used included anti-M16 (19924-1-AP, Proteintech), anti-IGF2BP1 (22803-1-AP, Proteintech), anti-IGF2BP2 (11601-1-AP, Proteintech), anti-IGF2BP3 (14642-1-AP, Proteintech), and anti-TM7SF2 (12033-1-AP, Proteintech). Staining intensity and the proportion of positive cells were assessed by two independent pathologists.

### *In vitro* functional assays

CCK-8, colony formation, and transwell assays were carried out as previously reported 17. For the CCK-8 assays, results for each group were calculated by averaging the values from five duplicate wells, with experiments repeated three times for statistical reliability. To ensure statistical accuracy, clone formation and transwell assays were done three times.

### Tumor xenograft model

This experiment was approved by the Institutional Animal Care and Use Committee of Nanjing Medical University. Five-week-old male athymic nude mice (BALB/c) were obtained from the Experimental Animal Center of Nanjing Medical University and randomly assigned to control and experimental groups, with five mice in each group. Each mouse was injected in the right axillary region with 100 µL of a solution containing 1 × 10^7^/mL SW480 cells. Every five days, tumor volumes were measured with calipers and computed using the method (V= width^2^ × length × 0.5). The mice were killed after four weeks, and the tumors were removed for weighing, immunohistochemical examination, and metabolite testing.

### RNA stability assay and protein stability detection

Cells were treated with 100 µg/ml of cycloheximide (CHX) to decrease protein translation and 10 µg/ml of actinomycin D (Act-D) to inhibit mRNA transcription. Cells were taken at particular times (0 h, 1 h, 2 h, 3 h, and 4 h). Total RNA was collected and examined by qRT-PCR to determine TM7SF2 mRNA stability. Cellular proteins were isolated and analyzed for TM7SF2 protein stability using Western blotting techniques.

### RIP assay

For RNA immunoprecipitation (RIP) assays, 3 µg of antibody was incubated with CRC cell lysate overnight. The following day, 50 µL of magnetic beads were added to the lysate, and the mixture was incubated for 2 hours. The eluted RNA complexes were analyzed by qRT-PCR.

### Quantitative detection of m6A RNA methylation (colorimetric method)

Total RNA from cells (shNC, shM16) was collected and assessed for quality. A Binding Solution was used to immobilize total RNA at the bottom of 96-well plates. The plates were treated with Capture Solution to bind m6A RNA, followed by Detection Solution to bind the Capture Solution. After incubating the 96-well plate with Developer Solution at room temperature and protected from light for 10 minutes, Stop Solution was added to terminate the enzymatic process once the solution turned medium blue. Absorbance at 450 nm was measured using an enzyme reader.

### Luciferase reporter assay

For luciferase reporter assays, cells (shNC and shM16) were transfected with reporter gene vectors using ReFcet (BIOG, China). Twenty-four hours post-transfection, firefly luciferase (Fluc) and Renilla luciferase (Rluc) activities were measured using the Dual-Luciferase Reporter Assay Kit (Vazyme, China) according to the manufacturer's protocol. The relative luciferase activity was calculated by determining the ratio of Fluc to Rluc activity, with results normalized for comparison.

### Metabolite detection

Intracellular lipid fluorescence detection was performed by fixing cells onto slides and staining them with Nile red dye. Lipids were visualized as orange or red under a fluorescence microscope.

For total cholesterol detection, 200 µL of isopropanol was added to homogenize either 1 × 10^6^ cells or 20 mg of tissue. According to the Amplex Red Cholesterol and Cholesteryl Ester Assay Kit (Beyotime Biotechnology, China) protocol, 10 µL of lysate was added to the prepared cholesterol detection solution and incubated at 37°C in the dark for 30 minutes. Absorbance at 570 nm was measured using a spectrophotometer to quantify cholesterol levels. The Amplex Red Free Fatty Acid (FFA) Assay Kit and Amplex Red Triglyceride Assay Kit (both from Beyotime Biotechnology, China) were also employed to measure FFA and triglyceride levels, respectively, using the same method.

### MeRIP-qPCR

Following the Magna MeRIP m6A Kit (Millipore, USA) instructions, RNA was fragmented to approximately 150 nt in length using Fragmentation buffer, then purified. Magnetic beads coated with anti-m6A antibody or IgG were pre-incubated for 30 minutes at room temperature to promote coupling, followed by a 2-hour incubation with fragmented RNA at 4°C. The eluted RNA complex was subjected to further qRT-PCR analysis.

### RNA pulldown

Full-length probes targeting TM7SF2 were developed and produced by Biotech. Biotinylated target RNA was then treated with magnetic beads to facilitate coupling. Beads were treated with cell lysate at 4°C for 2 hours, and the eluted RNA-protein complex was then examined by Western blotting.

### Statistical analysis

Statistical analyses were performed using GraphPad Prism 9.0 and SPSS version 23. The statistical tests applied included the two-tailed unpaired Student's t-test, one-way ANOVA, chi-square test, and nonparametric Wilcoxon rank sum test. Statistical significance was set at *p* < 0.05.

## Results

### Overexpression of M16 correlates with poor prognosis in CRC patients

**Figure 1:**
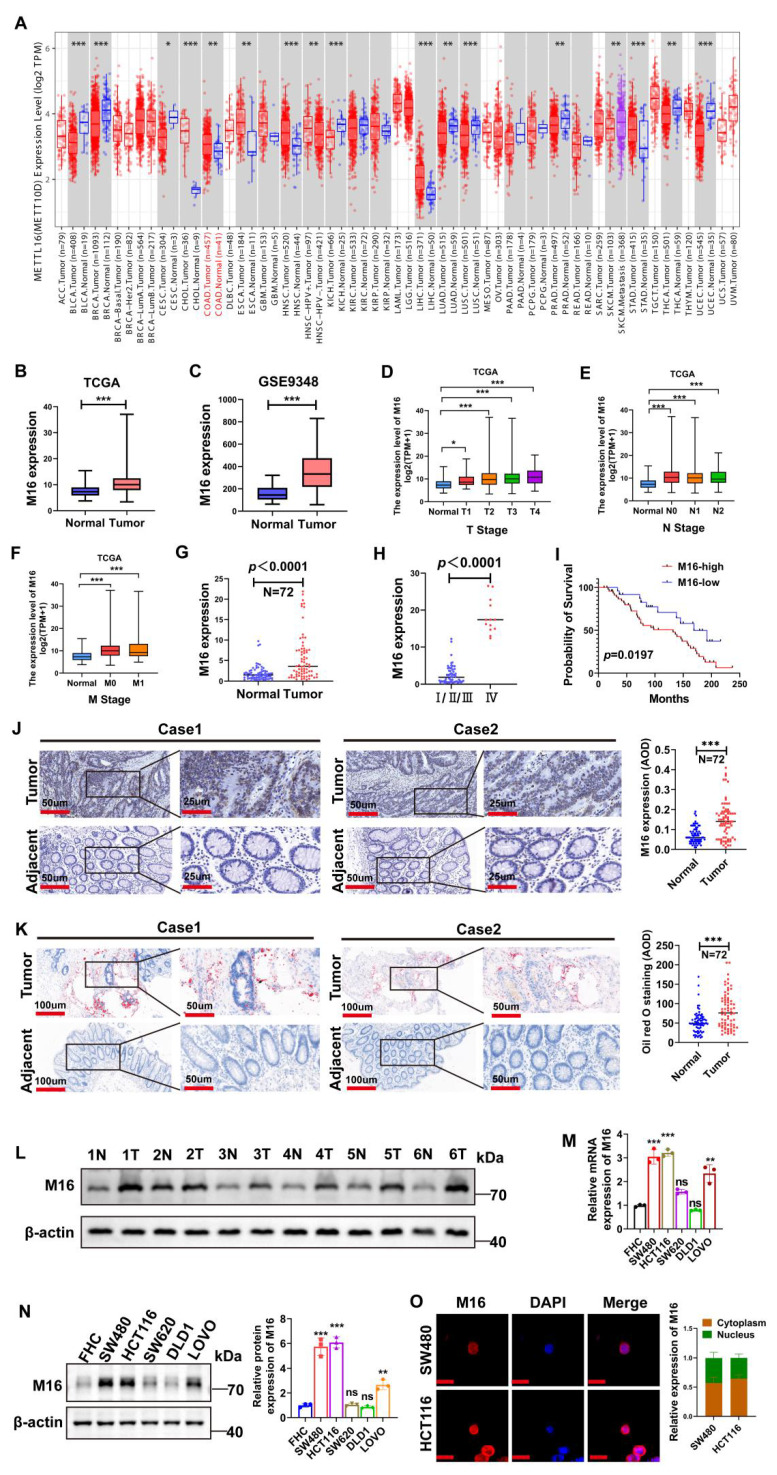
Increased M16 expression in CRC is associated with abnormal lipid metabolism and poor prognosis. A. Pan-cancer analysis of M16 was performed using the TIMER 2.0 database. B-C. M16 expression levels in the TCGA-CRC and GEO-CRC (GSE9348) datasets. D-F. Correlation between M16 expression and TNM staging in patients from the TCGA-CRC dataset. G. qRT-PCR was used to determine the expression levels of M16 in CRC tumors and neighboring non-tumor tissues. H. qRT-PCR study of M16 expression in CRC tissues categorized by pathological grade (I/II/III or IV). I. Kaplan-Meier survival curves comparing overall survival (OS) in patients with CRC exhibiting high or low M16 expression. J. IHC examination of M16 expression in CRC samples and neighboring non-tumor tissues. K. Oil Red O staining to examine lipid droplet distribution in CRC tissues and surrounding non-tumor tissues. L. Western blotting study of M16 expression in CRC tumors and nearby non-tumor tissues. M-N. M16 expression assessed by qRT-PCR and Western blotting in FHC, SW480, HCT116, SW620, DLD1, and LOVO cells. O. IF staining to determine M16 subcellular localization in SW480 and HCT116 cells. Scale bar: 10 μm. *p < 0.05, **p < 0.01, ***p < 0.001.

To investigate the role of m6A modifications in tumors, we conducted a bioinformatics analysis using RNA sequencing data from the TIMER 2.0 database, which integrates information from the TCGA database 18. The results revealed that M16 is upregulated across various cancers (Fig. 1A). M16 mRNA is highly overexpressed in CRC tissues relative to normal tissues, according to analysis of data from TCGA (Fig. 1B) and GEO (Fig. 1C). Further examination of TCGA data showed a significant positive correlation between M16 expression levels and advanced clinical stages, as well as distant metastasis in CRC patients (Fig. 1D-F). M16 mRNA levels were also examined in 72 matched CRC tissue samples, showing significant upregulation in cancer tissues (Fig. 1G). Additional analyses revealed that increased M16 levels were significantly associated with advanced pathological stages (IV) and poor survival outcomes (Fig. 1H, I). The findings of the IHC staining revealed that the CRC tissues had greater levels of M16 protein expression than the nearby non-tumor tissues (Fig. 1J).

The emerging field of metabolomics has underscored the importance of lipid reprogramming in CRC development and progression 19,20. Through Oil Red O staining, we analyzed lipid accumulation in CRC tissues and adjacent non-tumor tissues, finding more pronounced abnormal lipid deposition in CRC, particularly in tissues with high M16 expression (Fig. 1K). Further validation of M16 expression in several CRC tissue pairs was conducted using Western blotting (Fig. 1L). In subsequent experiments, M16 expression in various CRC cell lines was confirmed through qRT-PCR and Western blotting, showing significantly elevated mRNA and protein levels of M16 in most CRC cell lines compared to normal intestinal epithelial cells (Fig. 1M, N). Immunofluorescence (IF) experiments indicated that M16 is distributed in both the cytoplasm and nucleus of CRC cells (Fig. 1O). Overall, these results demonstrate that M16 is highly elevated in CRC and is closely associated with disease progression and poor prognosis.

### M16 promotes CRC progression *in vitro* and *in vivo*

**Figure 2:**
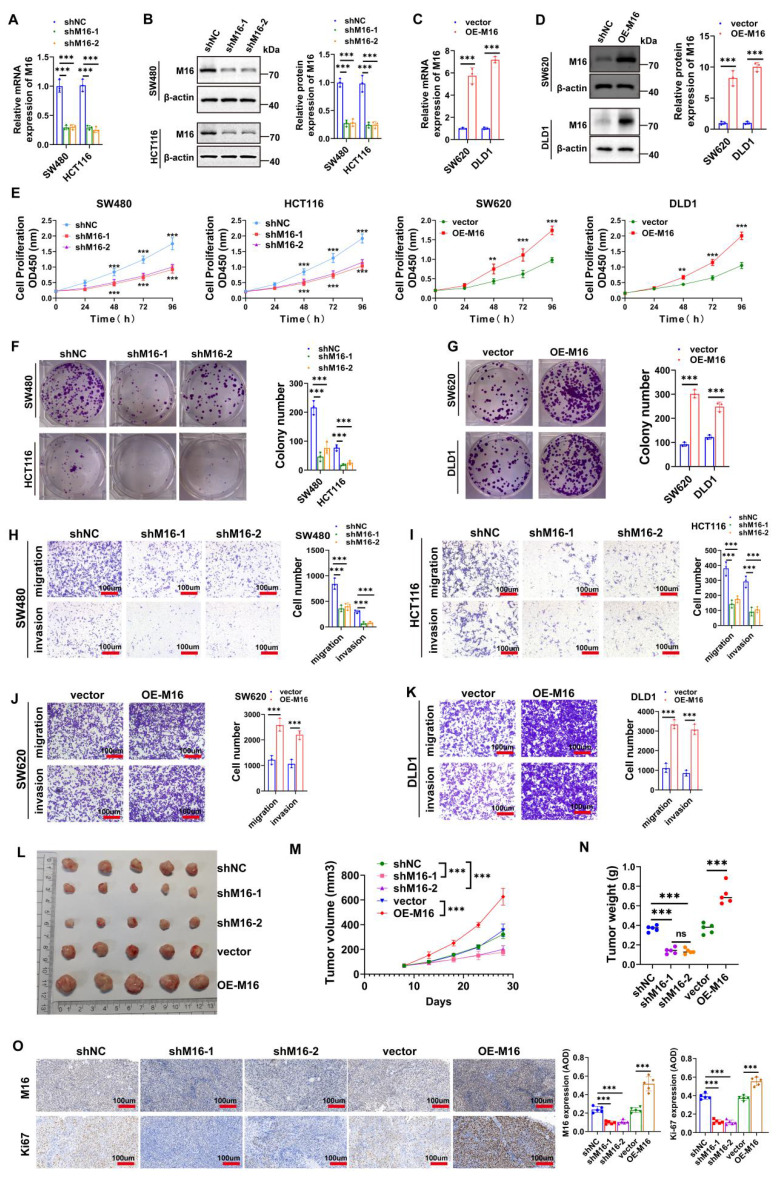
M16 promotes CRC progression *in vitro* and *in vivo*. A-D. qRT-PCR and Western blotting were used to confirm M16 knockdown and overexpression in CRC cells. E. CCK-8 assay was used to evaluate the proliferation capacity of CRC cells with M16 knockdown or overexpression. F-G. Colony formation ability of CRC cells with M16 knockdown or overexpression was assessed using plate colony formation assays. H-K. Migration and invasion capabilities of CRC cells with M16 knockdown or overexpression were analyzed by Transwell assays. L. Xenograft tumors from SW480-shNC, SW480-shM16-1, SW480-shM16-2, SW480-vector, and SW480-OE-M16. M. Growth curve of xenograft tumors was monitored and plotted over a specified period. N. Xenograft tumor weights were measured after euthanizing the mice. O. IHC analysis of M16 and Ki67 expression in xenograft tumors. *p < 0.05, **p < 0.01, ***p < 0.001.

High M16 expression in CRC is closely associated with disease progression and poor prognosis, suggesting its potential role as an oncogene in CRC. To investigate this further, SW480 and HCT116 cell lines, which exhibit notably high M16 expression, were selected. Two shRNAs (shM16-1 and shM16-2) were designed to knock down M16, while an overexpression plasmid was used to upregulate M16. The results revealed that shM16-1 and shM16-2 significantly reduced M16 mRNA and protein levels (Fig. 2A, B), while the overexpression plasmid markedly increased M16 expression in these CRC cells (Fig. 2C, D). M16 knockdown resulted in decreased proliferative, clone-forming, migratory, and invasive abilities of CRC cells, while M16 overexpression enhanced these properties (Fig. 2E-K). To assess the function of M16 *in vivo*, a xenograft tumor model was established. The results showed that M16 knockdown inhibited tumor growth, whereas M16 overexpression promoted tumor growth (Fig. 2L-N). Additionally, IHC staining revealed that Ki67 expression was reduced in the shM16-1 and shM16-2 groups, while it was elevated in the OE-M16 group (Fig. 2O). In summary, our findings indicate that M16 promotes the progression of CRC both *in vitro* and *in vivo*.

### M16 induces lipid metabolic reprogramming in CRC

**Figure 3:**
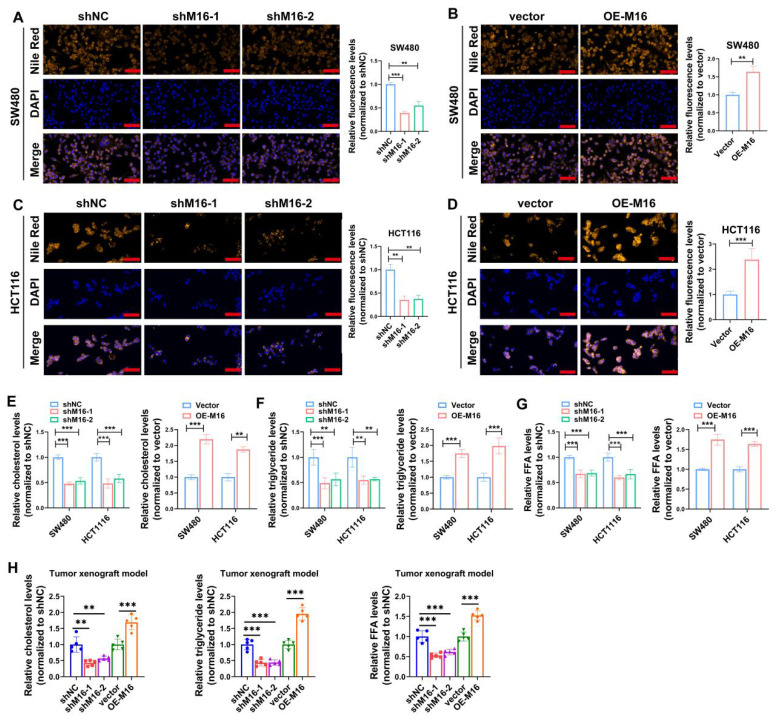
M16 promotes lipid metabolic reprogramming in CRC both *in vitro* and *in vivo*. A-D. In comparison to control groups, the expression of neutral lipids in SW480 and HCT116 with M16 overexpression or knockdown was examined using lipid fluorescence staining. Scale bar: 25 μm. E. Cholesterol expression was measured in SW480 and HCT116 cells that had M16 knockdown or overexpression. F. Triglyceride expression was measured using M16 overexpression or knockdown in SW480 and HCT116. G. M16 knockdown or overexpression was used to measure the expression of FFAs in SW480 and HCT116. H. Cholesterol, triglyceride, and FFA levels were measured in xenograft tumors from shNC, shM16-1, shM16-2, vector, and OE-M16 groups. *p < 0.05, **p < 0.01, ***p < 0.001.

Tumor growth and activity are energetically demanding, and the recent rise of metabolomics has highlighted the critical role of metabolic reprogramming in cancer development and progression 21,22. M16 facilitates glycolytic reprogramming and tumor progression in CRC 23. However, the relationship between M16 and lipid metabolic reprogramming in CRC remains poorly understood. Preliminary data indicate that high M16 expression in CRC tissues is linked to abnormal lipid deposition (Fig. 1K). To further investigate M16's role in lipid metabolic reprogramming, lipid fluorescence staining was performed. These experiments revealed that M16 knockdown in CRC cells resulted in reduced neutral lipid levels, while M16 overexpression significantly increased neutral lipid accumulation (Fig. 3A-D). Since neutral lipid droplets in the cytoplasm primarily consist of triglycerides, cholesterol, and FFAs, the levels of these metabolites were also measured in various CRC cell lines. The results indicated that M16 knockdown decreased triglycerides, cholesterol, and FFA levels, while M16 overexpression enhanced the expression of these metabolites (Fig. 3E-G). Similarly, in a xenograft tumor model, M16 silencing reduced the levels of cholesterol, triglycerides, and FFAs, while M16 overexpression led to an increase in these metabolites (Fig. 3H). These results confirm that M16 promotes lipid metabolic reprogramming in CRC.

### TM7SF2 is a critical downstream gene of M16 in CRC cells

**Figure 4:**
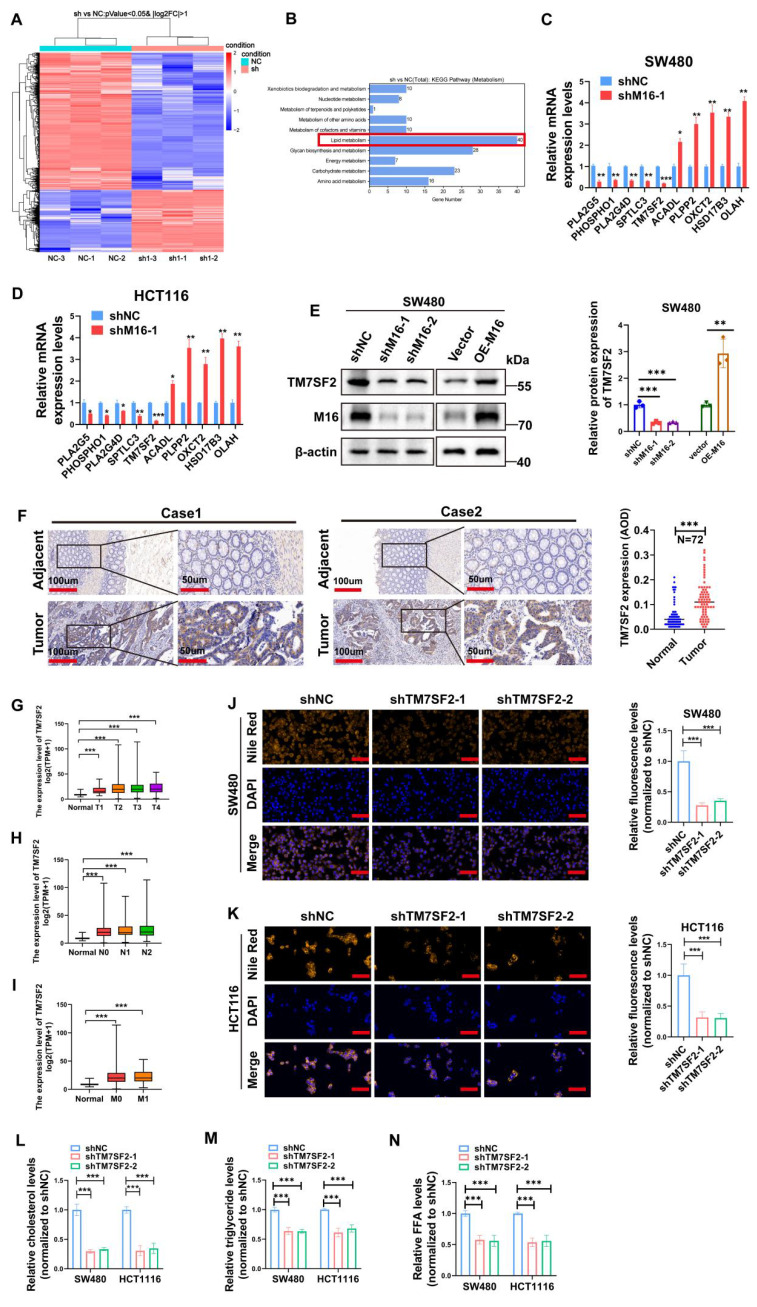
TM7SF2 induces lipid metabolic reprogramming in CRC cells. A. Heatmap displaying differentially expressed genes in SW480 cells following M16 knockdown. B. KEGG enrichment analysis identifying metabolic pathways associated with M16. C-D. qRT-PCR analysis identifying lipid metabolism-related genes with significant differential expression in SW480 and HCT116 cells following M16 knockdown. E. Western blotting confirming TM7SF2 protein expression in SW480 cells following M16 overexpression or knockdown. F. IHC labeling to examine the TM7SF2 protein expression in neighboring non-tumor tissues and CRC tissues. G-I. Correlation between TM7SF2 expression and TNM staging in TCGA-CRC patients. J-K. Lipid fluorescence staining assessing neutral lipid expression in CRC cells (SW480 and HCT116) with TM7SF2 knockdown compared to control groups. Scale bar: 25 μm. L-N. Assessment of cholesterol, triglycerides, and FFAs in CRC cells with TM7SF2 knockdown. *p < 0.05, **p < 0.01, ***p < 0.001.

To elucidate the molecular mechanisms underlying M16's role in lipid metabolic reprogramming in CRC, RNA sequencing analysis was performed to identify downstream genes regulated by M16. Using a threshold of *p* < 0.05 and |log FC| > 1, 1265 differentially expressed genes (DEGs) were identified (Fig. 4A). Gene enrichment analysis revealed significant associations between these DEGs and various metabolic pathways, with lipid metabolism being the most prominent (Fig. 4B). Among the M16-regulated DEGs, 40 were closely associated with lipid metabolism. Ten genes exhibiting the most significant differential expression were selected for further investigation.

qRT-PCR validation in SW480 and HCT116 cell lines demonstrated that TM7SF2 expression was most notably reduced following M16 knockdown (Fig. 4C, D). Western blotting confirmed that M16 silencing suppressed TM7SF2 protein expression, whereas M16 overexpression increased it (Fig. 4E). IHC analysis revealed significant upregulation of TM7SF2 in CRC tissues compared to adjacent non-tumor tissues (Fig. 4F). Further analysis of TCGA-CRC data demonstrated a strong positive correlation between TM7SF2 expression and advanced clinical stages, lymph node metastasis, and distant metastasis in patients with CRC (Fig. 4G-I). As a key enzyme in cholesterol synthesis, TM7SF2 has been implicated in lipid metabolic reprogramming and the progression of cervical cancer 24. However, its specific role in CRC progression remains unclear and warrants further investigation. Lipid fluorescence staining revealed that silencing TM7SF2 significantly reduced neutral lipid content in CRC cells (Fig. 4J, K). Metabolite analysis showed a reduction in triglycerides, cholesterol, and FFAs in TM7SF2-silenced CRC cells (Fig. 4L-N). These results suggest that TM7SF2, as a downstream target of M16, contributes to lipid metabolic reprogramming in CRC cells.

### M16 promotes TM7SF2 mRNA stability through m6A-dependent mechanism

**Figure 5:**
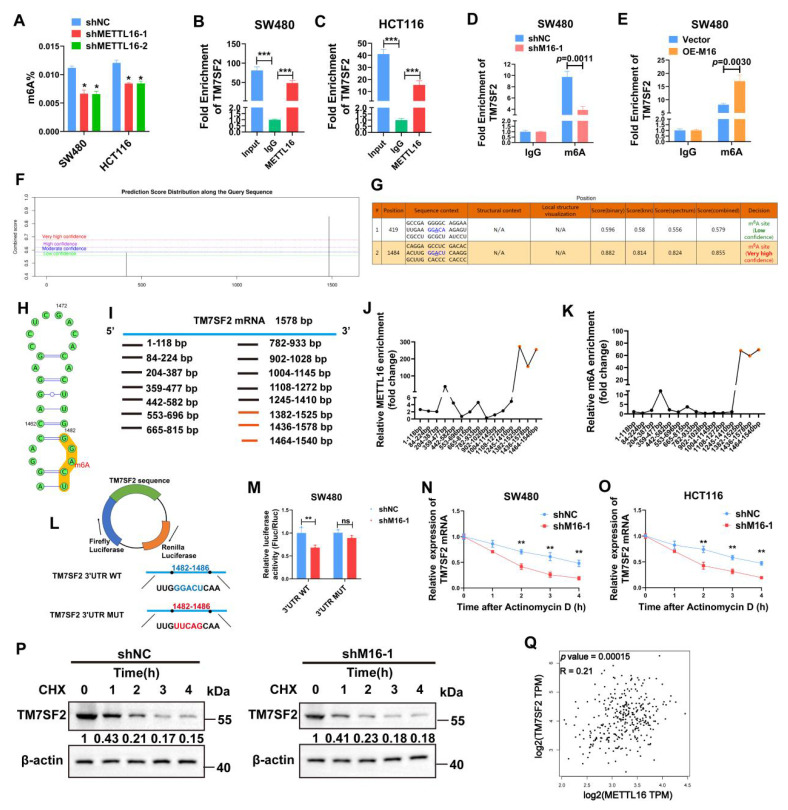
M16 promotes TM7SF2 mRNA stability through m6A-dependent mechanisms. A. Total m6A levels were assessed to determine the impact of M16 knockdown in SW480 and HCT116 cells. B-C. RIP-qPCR experiments identified whether TM7SF2 mRNA binds with M16 in SW480 and HCT116 cells. D-E. RIP-qPCR was conducted to evaluate m6A enrichment on TM7SF2 mRNA after M16 knockdown or overexpression in SW480 cells. F-G. SRAMP website predicts possible m6A modification sites on TM7SF2 mRNA. H. Secondary structure prediction of TM7SF2 mRNA containing m6A sites. I. Primers were designed for specific TM7SF2 mRNA fragments. J-K. TM7SF2 mRNA was treated with metal ions to fragment it into 100-200 bp lengths, followed by M16-RIP-PCR to identify specific binding sites of M16 on TM7SF2 mRNA in SW480 cells. L. Creation of luciferase reporter plasmids encoding firefly and renilla luciferases. The 3' UTR of firefly luciferase was inserted with either wild-type (WT) or mutant (Mut) sequences. M. Transfection of constructed luciferase reporter plasmids into SW480 cells and assessment of firefly and renilla luciferase activities. N-O. Treatment of SW480 and HCT116 cells with actinomycin D (10 µg/ml) post M16 knockdown, and assessment of TM7SF2 mRNA stability at specific times (0h, 1h, 2h, 3h, 4h) via qRT-PCR. P. Treatment of SW480 cells post M16 knockdown with CHX (100 µg/ml), and monitoring of TM7SF2 protein stability at specific times (0h, 1h, 2h, 3h, 4h) via Western blotting. Q. Correlation between M16 mRNA and TM7SF2 mRNA. *p < 0.05, **p < 0.01, ***p < 0.001.

M16, acting as an RNA methyltransferase, plays a pivotal role in RNA splicing, translocation, stability, and protein translation, influencing various cancer progressions 25,26. To investigate its function in CRC, the change in total m6A levels was assessed following M16 knockdown. The findings revealed a substantial drop in total m6A levels in CRC cells of the shM16 group compared to the shNC group, highlighting M16's crucial involvement in m6A alteration in CRC (Fig. 5A). To determine whether M16 directly or indirectly affects TM7SF2 mRNA expression, RIP was performed to examine if M16 protein directly binds to TM7SF2 mRNA. RIP-qPCR results confirmed a direct interaction between M16 protein and TM7SF2 mRNA (Fig. 5B, C). Further analysis of m6A levels on TM7SF2 mRNA using meRIP revealed that M16 knockdown decreased, while overexpression of M16 increased, m6A levels on TM7SF2 mRNA (Fig. 5D, E).

To explore potential m6A modification sites on TM7SF2 mRNA, this study utilized the SRAMP website 27, which predicted two potential m6A sites, particularly at the 1484bp region (Fig. 5F-H). To identify the specific m6A site, the mRNA was treated with metal ions to fragment it into 100-200 bp lengths. M16-RIP-qPCR analysis revealed the strongest binding between M16 and the TM7SF2 mRNA fragment from 1464-1540bp (Fig. 5I, J). Similarly, meRIP-qPCR showed the highest m6A enrichment at this same fragment (1464-1540bp) (Fig. 5K), corroborating the SRAMP predictions. Additionally, luciferase reporter plasmids for both wild-type and mutant TM7SF2 were constructed to examine M16's effect on TM7SF2 mRNA (Fig. 5L). The results demonstrated that M16 knockdown significantly suppressed luciferase activity in the TM7SF2 WT group compared to the MUT group (Fig. 5M). Since m6A modification impacts RNA stability, thus influencing target RNA expression 28,29, RNA stability assays and protein stability tests confirmed that M16 knockdown reduced the stability of TM7SF2 mRNA without affecting the stability of TM7SF2 protein (Fig. 5N-P). Further analysis of TCGA sequencing data revealed a positive correlation between M16 and TM7SF2 mRNA expression levels in CRC (Fig. 5Q) 30, consistent with our experimental findings. In summary, these results suggest that M16 enhances TM7SF2 expression by promoting the stability of TM7SF2 mRNA, but not its protein stability.

### M16 modulates TM7SF2 m6A modification *via* m6A readers IGF2BP1 and IGF2BP2

**Figure 6:**
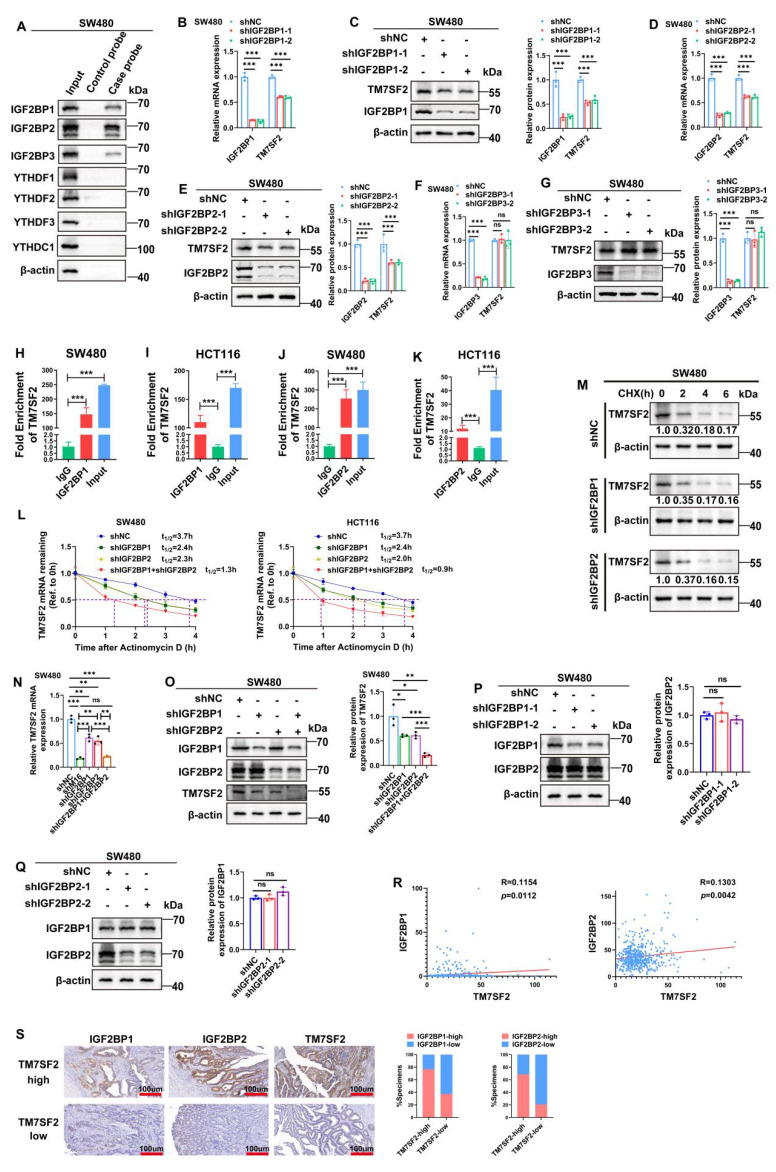
TM7SF2 mRNA is recognized by IGF2BP1 and IGF2BP2. A. RNA pulldown with biotinylated TM7SF2 as the probe and beads alone as the control probe, followed by Western blotting, identified the readers interacting with TM7SF2 mRNA in SW480 cells. B-C. Western blotting and qRT-PCR confirmed the expression of IGF2BP1 and TM7SF2 mRNA and protein levels following IGF2BP1 knockdown. D-E. Western blotting and qRT-PCR verified the expression of IGF2BP2 and TM7SF2 mRNA and protein following IGF2BP2 knockdown. F-G. qRT-PCR and Western blotting were used to confirm the expression of IGF2BP3 and TM7SF2 mRNA and protein following IGF2BP3 knockdown. H-I. RIP-PCR showed that IGF2BP1 binds to TM7SF2 mRNA in SW480 and HCT116. J-K. RIP-PCR showed that IGF2BP2 binds to TM7SF2 mRNA in SW480 and HCT116. L. Treatment of CRC cells with actinomycin D (10 µg/ml) and measurement of TM7SF2 mRNA stability at specified times (0h, 1h, 2h, 3h, 4h) in shNC, shIGF2BP1, shIGF2BP2, and shIGF2BP1+shIGF2BP2 groups via qRT-PCR. M. Treatment of CRC cells with CHX (100 µg/ml) and measurement of TM7SF2 protein stability at specified times (0h, 1h, 2h, 3h, 4h) in shNC, shIGF2BP1, shIGF2BP2 groups via qRT-PCR. N. qRT-PCR experiments were conducted to assess the expression levels of TM7SF2 mRNA following the knockdown of M16, IGF2BP1, or IGF2BP2. O. Western blotting assays were conducted to measure TM7SF2 protein expression in the shIGF2BP1, shIGF2BP2, and shIGF2BP1+shIGF2BP2 groups. P. After knocking down IGF2BP1, Western blotting was performed to measure IGF2BP2 protein expression. Q. Following IGF2BP2 knockdown, Western blotting was utilized to assess IGF2BP1 protein expression. R. Correlation between TM7SF2 mRNA and IGF2BP1 mRNA or IGF2BP2 mRNA. S. Using IHC to analyze the correlation between TM7SF2 protein and IGF2BP1 protein or IGF2BP2 protein. *p < 0.05, **p < 0.01, ***p < 0.001.

In the dynamic regulation of mRNA expression *via* m6A modification, the writers or erasers introduce m6A modifications at specific sites on mRNA, and readers recognize these sites to stabilize the mRNA 31,32. To identify the readers directly binding to TM7SF2 mRNA, RNA pulldown experiments were performed. The results revealed that IGF2BP1, IGF2BP2, and IGF2BP3 interact directly with TM7SF2 mRNA (Fig. 6A). Notably, knockdown of IGF2BP1 and IGF2BP2 led to a reduction in both TM7SF2 mRNA and protein levels (Fig. 6B-E), while knockdown of IGF2BP3 had no impact on their expression (Fig. 6F, G). RIP-qPCR experiments demonstrated that TM7SF2 mRNA directly binds to IGF2BP1 and IGF2BP2 proteins in both SW480 and HCT116 cells (Fig. 6H-K).

Previous reports indicate that IGF2BP1 and IGF2BP2 function as m6A-dependent readers, regulating mRNA stability 33,34. Therefore, RNA stability assays were conducted, confirming that knockdown of IGF2BP1 or IGF2BP2 individually decreased TM7SF2 mRNA stability, and simultaneous knockdown of both resulted in an even faster decay of TM7SF2 mRNA (Fig. 6L). Protein stability assays showed that compared to the shNC group, knockdown of IGF2BP1 or IGF2BP2 did not alter TM7SF2 protein stability (Fig. 6M). These results suggest that IGF2BP1 and IGF2BP2 enhance the expression level of TM7SF2 mRNA by affecting its stability.

Furthermore, to explore whether IGF2BP1 and IGF2BP2 together influence the expression level of TM7SF2 mRNA, we treated CRC cells with both shIGF2BP1 and shIGF2BP2. The results revealed that knockdown of either IGF2BP1 or IGF2BP2 alone led to a reduction in TM7SF2 mRNA expression. Interestingly, TM7SF2 mRNA expression was lower in the shM16 and shIGF2BP1+IGF2BP2 groups than in the individual shIGF2BP1 or shIGF2BP2 groups (Fig. 6N). Similarly, Western blotting studies revealed lower protein levels of TM7SF2 in the shIGF2BP1+shIGF2BP2 group than in the shIGF2BP1 or shIGF2BP2 group (Fig. 6O). Additionally, Western blotting experiments demonstrated that knockdown of either IGF2BP1 or IGF2BP2 did not affect the expression of the other reader (Fig. 6P, Q). Additional examination of the TCGA sequencing data showed that IGF2BP1 or IGF2BP2 was favorably linked with the expression level of TM7SF2 mRNA in CRC, which is in line with our experimental findings (Fig. 6R). IHC staining also revealed that CRC tissues with high TM7SF2 expression typically exhibited elevated levels of IGF2BP1 and IGF2BP2 (Fig. 6S). In summary, these results suggest that IGF2BP1 and IGF2BP2 synergistically enhance the stability and expression of TM7SF2 mRNA *via* M16-mediated m6A modification.

### M16 facilitates lipid metabolic reprogramming and CRC progression through regulation of TM7SF2

**Figure 7:**
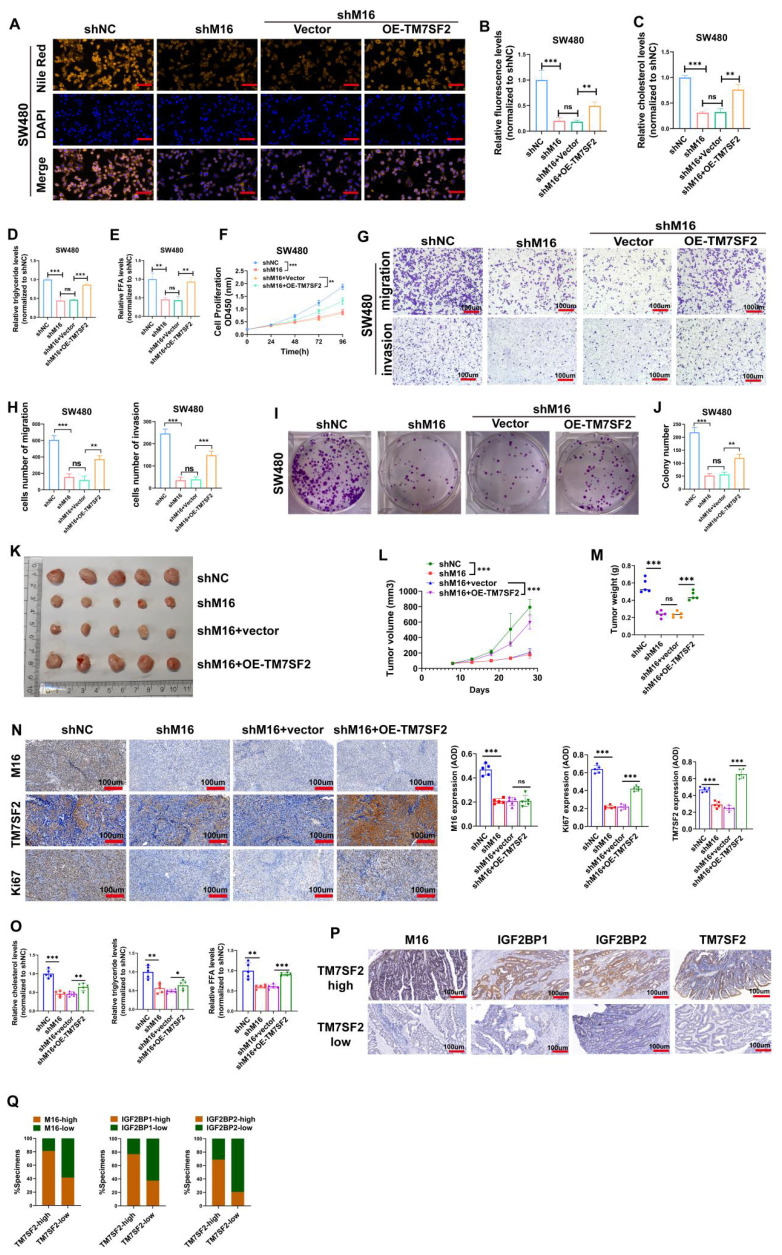
M16/m6A/TM7SF2 axis promotes lipid metabolic reprogramming and CRC progression. A-B. Lipid fluorescence assays evaluated the effect of TM7SF2 overexpression on neutral lipid levels in SW480 cells after M16 knockdown, with quantitative analysis of the results. Scale bar: 25 µm. C-E. The impact of TM7SF2 overexpression on cholesterol, triglycerides, and FFA levels in SW480 cells following M16 knockdown was measured. F. CCK-8 assays were conducted to assess how TM7SF2 overexpression affected SW480 cell proliferation after M16 knockdown. G-H. The effect of TM7SF2 overexpression on the migratory and invasive potential of SW480 cells after M16 knockdown was evaluated using Transwell assays. I-J. Colony formation assays evaluated how TM7SF2 overexpression affected SW480 cells' colony-forming capacity after M16 knockdown. K. Xenograft tumors from SW480-shNC, SW480-shM16, SW480-shM16+vector, and SW480-shM16+OE-TM7SF2. L. Growth curves of xenograft tumors were monitored and plotted over a specific period. M. Xenograft tumor weights were measured after the mice were euthanized. N. IHC analysis of M16, TM7SF2, and Ki67 expression in xenograft tumors. O. Cholesterol, triglycerides, and FFA expression were measured in xenograft tumors from shNC, shM16, shM16+vector, and shM16+OE-TM7SF2 groups. P-Q. IHC analysis of the correlation between TM7SF2 protein and M16, IGF2BP1, or IGF2BP2 proteins. Scale bar: 100 µm. *p < 0.05, **p < 0.01, ***p < 0.001.

Experimental results confirmed TM7SF2 as a critical downstream target of M16. We conducted rescue experiments to validate the key mediatory role of TM7SF2 in M16-induced lipid metabolic reprogramming. Lipid fluorescence assays indicated that, compared to the shNC group, lipid content significantly decreased in the shM16 group, but overexpression of TM7SF2 reversed the reduction caused by M16 knockdown (Fig. 7A, B). Intracellular metabolite analysis revealed decreased levels of cholesterol, triglycerides, and FFAs in CRC cells following M16 knockdown, with overexpression of TM7SF2 mitigating these effects (Fig. 7C-E). These results suggest that M16 drives lipid metabolic reprogramming in CRC through TM7SF2 regulation.

Additionally, to confirm the possible functional significance of the M16/m6A/TM7SF2 axis in CRC development, we employed *in vitro* functional assays and animal models. *In vitro* assays showed that M16 knockdown inhibited CRC cell proliferation, colony formation, migration, and invasion, while TM7SF2 overexpression restored these capabilities (Fig. 7F-J). In vivo experiments revealed that M16 knockdown suppressed tumor growth, while TM7SF2 overexpression reversed this effect (Fig. 7K-N). Additionally, TM7SF2 overexpression alleviated the reduction in cholesterol, triglycerides, and FFAs observed in xenograft tumors after M16 knockdown (Fig. 7O). IHC staining analysis showed that CRC tissues with high TM7SF2 expression also typically exhibit high levels of M16, IGF2BP1 and IGF2BP2 (Fig. 7P, Q). In conclusion, these results indicate that M16 promotes lipid metabolic reprogramming and CRC progression by regulating TM7SF2.

## Discussion

CRC, as one of the most common malignancies, poses a significant threat to human health 1. The rising incidence and mortality associated with CRC highlight the urgent need for novel biomarkers and effective therapeutic targets. The emergence of metabolomics has underscored the pivotal role of energy reprogramming in CRC development 35-37. In our study, we found that the m6A methyltransferase M16 is significantly overexpressed in CRC tissues and is associated with poor patient outcomes. M16 enhances CRC proliferation and migration abilities both *in vitro* and *in vivo* and induces lipid metabolic reprogramming in CRC cells. Mechanistically, M16 facilitates the m6A modification of TM7SF2 mRNA, leading to its recognition, stabilization, and upregulation by IGF2BP1 and IGF2BP2. Notably, IGF2BP1 and IGF2BP2 not only increase TM7SF2 mRNA stability independently, but their combined actions lead to a more substantial stabilization of TM7SF2 mRNA. These findings suggest that the M16/m6A/TM7SF2 axis accelerates CRC progression.

Metabolomics has revealed the critical role of metabolic reprogramming in CRC 38,39. Dysregulated lipid metabolism drives tumor cell proliferation and promotes liver metastasis, exacerbating patient prognosis 40-42. M16 contributes to CRC progression by enhancing glycolytic reprogramming, maintaining chromosomal stability, and facilitating immune evasion 23,43,44. However, the connection between M16 and lipid metabolism in CRC has not been explored until now. In this study, we observed a higher number of lipid droplets in CRC tissues with high M16 expression, suggesting a potential connection between M16 and lipid metabolic reprogramming. Lipid fluorescence assays revealed that high M16 expression increases the content of neutral lipids, including cholesterol, triglycerides, and FFAs. Further analysis confirmed that M16 upregulates the expression of cholesterol, triglycerides, and FFAs, thus influencing CRC lipid metabolic reprogramming.

RNA sequencing and meRIP-qPCR identified TM7SF2 as a key target in M16-induced lipid metabolic reprogramming. M16 mediates m6A modification of TM7SF2 mRNA, enhancing its stability and promoting TM7SF2 expression. TM7SF2, a key enzyme in cholesterol biosynthesis, plays a vital role in lipid metabolic processes 45. Liu *et al.* demonstrated that upregulated TM7SF2 contributes to lipid droplet formation and increased fatty acid content in cervical cancer cells, promoting carcinogenesis 24. However, the biological functions of TM7SF2 in CRC remained unexplored. In the present study, TM7SF2 is overexpressed in CRC, and its overexpression can rescue the reduction in cholesterol, triglycerides, and FFAs caused by M16 silencing. These results suggest that TM7SF2 is a critical mediator in M16-regulated lipid metabolic reprogramming in CRC.

M16, in conjunction with recognition proteins, enhances the stability of target mRNAs. M16 regulates SYNPO2L mRNA stability through its interaction with YTHDC1, promoting tumor cell epithelial-mesenchymal transition (EMT) and infiltration of tumor-associated fibroblasts 46. Additionally, M16 upregulates MYBL2 mRNA stability in an IGF2BP1-dependent manner, influencing the cell cycle during early embryonic development 47. These findings highlight the critical function of readers in maintaining the stability of TM7SF2 mRNA, which is controlled by M16. RNA pulldown and RIP-qPCR analyses revealed that IGF2BP1 and IGF2BP2 bind to and stabilize TM7SF2 mRNA. Interestingly, compared to the substantial reduction in TM7SF2 mRNA following M16 knockdown, silencing either IGF2BP1 or IGF2BP2 alone led to a mild inhibition of TM7SF2 mRNA expression. Additionally, it has been reported that IGF2BP1, IGF2BP2, and IGF2BP3 work together to stabilize MYC mRNA, enhancing its carcinogenic effects 48,49. This suggests that target mRNAs may be regulated not only by a single reader but also by multiple readers acting in concert. However, the cooperative upregulation of target mRNA expression by M16 with multiple readers has not been previously reported. Based on existing literature and our experimental results, IGF2BP1 and IGF2BP2 may work together to upregulate TM7SF2 mRNA expression in an additive manner. Further studies found that compared to the shIGF2BP1 or shIGF2BP2 groups, the stability and expression levels of TM7SF2 mRNA in the combined shIGF2BP1+shIGF2BP2 group were significantly reduced. These findings confirm our hypothesis that IGF2BP1 and IGF2BP2 cooperate to enhance TM7SF2 expression through M16-mediated m6A modification.

## Conclusions

**Figure 8:**
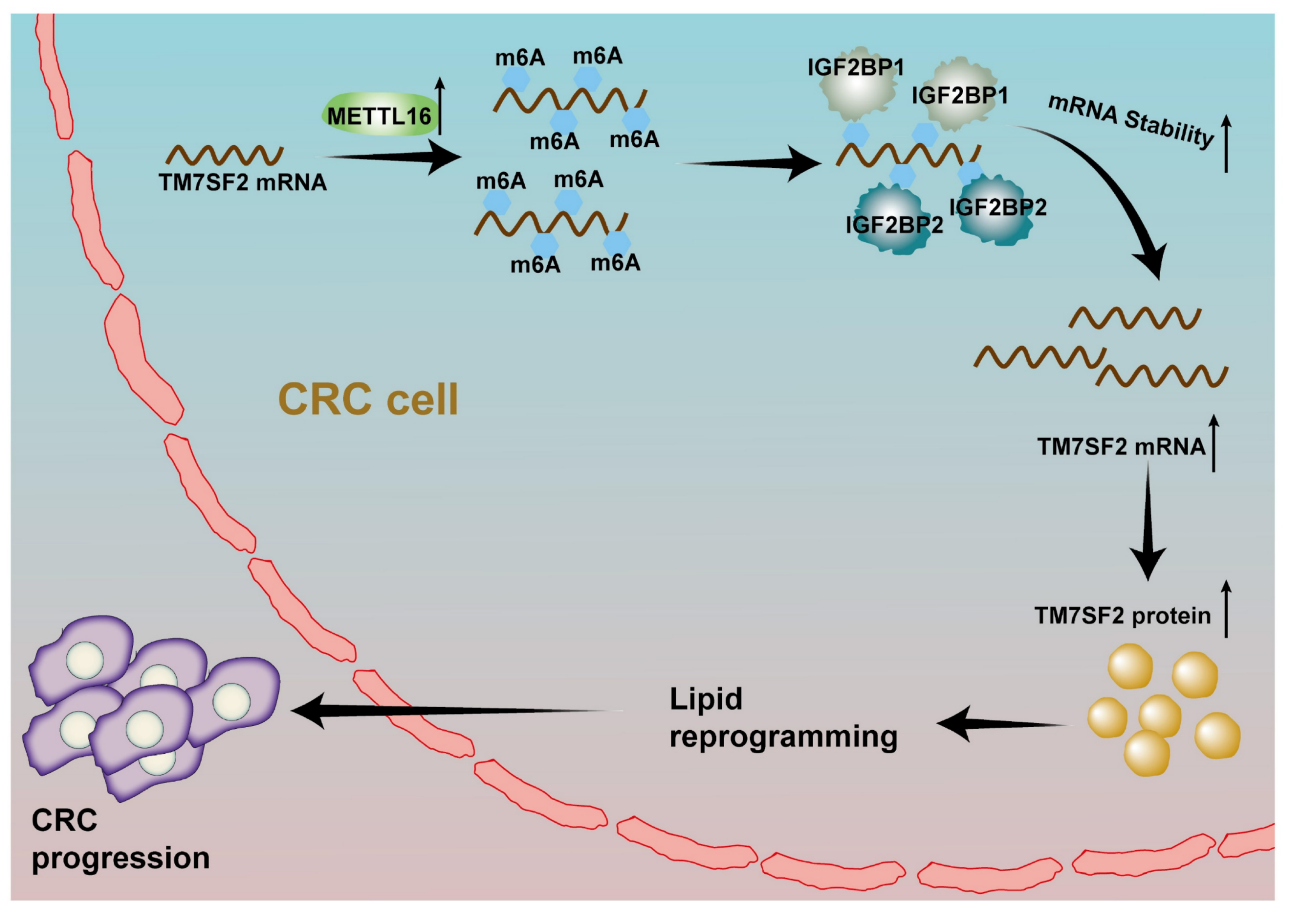
A schematic illustration describes how M16 overexpression in CRC promotes m6A modification of TM7SF2 mRNA, leading to enhanced stability of TM7SF2 mRNA by IGF2BP1 and IGF2BP2. Increased TM7SF2 promotes lipid reprogramming and CRC progression.

In conclusion, this study reveals that upregulated M16 promotes lipid metabolic reprogramming and CRC progression, correlating with poor patient outcomes. Mechanistically, M16 mediates m6A modification to upregulate TM7SF2 expression in an IGF2BP1- and IGF2BP2-dependent manner (Fig. 8). These findings uncover novel mechanisms in M16-mediated CRC lipid metabolic reprogramming, offering new insights into potential biomarkers and therapeutic targets for CRC.

## Supplementary Material

Supplementary tables.
