# Supplementary material for: METTL16 Promotes Lipid Metabolic Reprogramming and Colorectal Cancer Progression
Source: Int J Biol Sci. 2025 Jul 24;21(11):4782–97. doi: 10.7150/ijbs.105391 (PMC12374814; doi:10.7150/ijbs.105391)
Supplement: Supplementary file 1 — Supplementary tables. [file ijbsv21p4782s1.pdf]

Table S1. The sequences of siRNAs or shRNAs.

Oligonucleotides	Sequences
scrambled siRNA	UUCUCCGAACGUGUCACGUTT
shIGF2BP2-1	CAUGCCGCAUGAUUCUUGATT
shIGF2BP2-2	GAACGAACUGCAGAACUUATT
shIGF2BP1-1	CCGGGAGCAGACCAGGCAATT
shIGF2BP1-2	UGAAUGGCCACCAGUUGGATT
shIGF2BP3-1	CGGUGAAUGAACUUCAGAATT
shIGF2BP3-2	GCAAAGGAUUCGGAAACTT
shTM7SF2-1	CUGUUUCUUCGACUUCAAAUA
shTM7SF2-2	GCGAAUUCCCAGAAAAACACU
shMETTL16-1	ATGGCTGGTATTTCCTCGCAA
shMETTL16-2	GGAAGATTTTGGACTTTCT

Table S2. Primers used in the study.

Primers	Sequences（5'-3'）
METTL16 forward primer	TGAGAGGTGGCTGTTGGTC
METTL16 reverse primer	AGTGAGCTAAGATCGCACCA
TM7SF2 forward primer	CCGCCATCCCAACTATCTT
TM7SF2 reverse primer	GGAGCCGCTTCAGTAGATGT
IGF2BP1 forward primer	TAGTACCAAGAGACCAGACCC
IGF2BP1 reverse primer	GATTTCTGCCCGTTGTTGTC
IGF2BP2 forward primer	CTACGCCTTCGTGGACTACC
IGF2BP2 reverse primer	CATCCAACACCTCCCACTG
IGF2BP3 forward primer	TTGCAGGAATTGACGCTGTA
IGF2BP3 reverse primer	ACCCAAGGCGTTCAGATTTA
PLA2G5 forward primer	ACGGCTTCTACGGCTGTTAC
PLA2G5 reverse primer	ATGTTGCAGCCCTTCTCCTC
PHOSPHO1 forward primer	ATTTCCTGGTCCTCCCAACT
PHOSPHO1 reverse primer	GCCATAGGTGGAGACCCTCT
PLA2G4D forward primer	ACCAGTCATCCTGTGTGGAA
PLA2G4D reverse primer	GGGCGATCTGACGTTTCTT
SPTLC3 forward primer	GCAGACCCTTTCAGGGATTC
SPTLC3 reverse primer	TACCCTCCTGCCTGGAACTT
ACADL forward primer	CCCAGGATACCGCAGAACTA
ACADL reverse primer	TTGCACTGTCTGTAGGTGAGC
PLPP2 forward primer	TACATGATTGGGCGTCTGAG
PLPP2 reverse primer	CCCACTTCCAACAGAGTCGT
OXCT2 forward primer	GGGCAGAAATACGAGAAACG
OXCT2 reverse primer	GGATGCCTATGCCCAGATT
HSD17B3 forward primer	GAGATCGAGCGGACTACAGG
HSD17B3 reverse primer	AATGGCTTGGGAGAAGGTTT
OLAH forward primer	ACGAGGTCAGGAGATCGAGA
OLAH reverse primer	GGTTCACTGCAAGCTCCAC
TM7SF2 1-118bp forward primer	GTCTGCGTTCCGTGTCCAGG
TM7SF2 1-118bp reverse primer	GCGCTCACAATAGTCAGTCAAGGA
TM7SF2 84-224bp forward primer	CGGACAGTGTTTCCTTGACTGAC
TM7SF2 84-224bp reverse primer	GGCAGCAGCAGTAGCAGAG
TM7SF2 204-387bp forward primer	GGCTCTGCTACTGCTGCTG
TM7SF2 204-387bp reverse primer	CGCCGGCAGTAGGTAGAG
TM7SF2 359-477bp forward primer	TGCAGGCGGCGCTCTACCTAC

TM7SF2 359-477bp reverse primer	CAGGGCTGTCAGCACCAGG
TM7SF2 442-582bp forward primer	ATTAACGGCTTCCAGGCCCTG
TM7SF2 442-582bp reverse primer	GAGGCTGAAGATGAAAGCGGTG
TM7SF2 553-696bp forward primer	GCCACCCTCACCGCTTTCA
TM7SF2 553-696bp reverse primer	ACAGATACGAGGGTTGAGCTCT
TM7SF2 665-815bp forward primer	TTCTGGGACGAGAGCTCAAC
TM7SF2 665-815bp reverse primer	CACATGGCCAGTGAGGGACT
TM7SF2 782-933bp forward primer	CAGAGCTTCGAGGCAGTCC
TM7SF2 782-933bp reverse primer	GTCCCCAAACGCCAGCATGAA
TM7SF2 902-1028bp forward primer	ACGGGTTTGGCTTCATGCTG
TM7SF2 902-1028bp reverse primer	AGGCAGATGACAGAGGCCATG
TM7SF2 1004-1145bp forward primer	TGCCCATGGCCTCTGTCATCT
TM7SF2 1004-1145bp reverse primer	CCTGTGGCTGTAGAGATGGTC
TM7SF2 1108-1272bp forward primer	AGAGTGGCTGGGCTTGAGAC
TM7SF2 1108-1272bp reverse primer	GAGGTAGAAGTAGGGCAGCAGG
TM7SF2 1245-1410bp forward primer	GTCACACCTGCTGCCCTACTT
TM7SF2 1245-1410bp reverse primer	GGAGCCGCTTCAGTAGATGTAGG
TM7SF2 1382-1525bp forward primer	TCATGCCCTACATCTACTGAAGC
TM7SF2 1382-1525bp reverse primer	GTTGTTCATCCTCAGGGCTGG
TM7SF2 1436-1578bp forward primer	CACTCATCCACCAGCACACC
TM7SF2 1436-1578bp reverse primer	CCTTGCTCTAAACCACCTCTTCT
TM7SF2 1464-1540bp forward primer	GGAGCCTCGACACACTTGG
TM7SF2 1464-1540bp reverse primer	CACCTCTTCTCTGAGGTTGTTCA
β-actin forward primer	CATGTACGTTGCTATCCAGGC
β-actin reverse primer	CTCCTTAATGTCACGCACGAT
GAPDH forward primer	GGAGCGAGATCCCTCCAAAAT
GAPDH reverse primer	GGCTGTTGTCATACTTCTCATGG

Table S3. METTL16 expression in clinical and pathological characteristics of colorectal cancer patients.

**Variable**	**Expression of METTL16 in colorectal cancer**			***P* value**
	**Low （n=24）**	**High （n=48）**	**Total (n=72)**	
**Age**				
≤65	16	13	29	0.01
＞65	8	35	43	
**Gender**				
Female	13	21	34	0.404
Male	11	27	38	
**Maximum tumor size**				
<4 cm	16	15	31	0.004
⩾4 cm	8	33	41	
**T stage**				
T1 and T2	15	6	21	< 0.001
T3 and T4	9	42	51	
**N stage**				
N0	16	11	27	< 0.001
N1 and N2	8	37	45	
**Tumor stage**				
I and II	16	11	27	< 0.001
III and IV	8	37	45	
